# Predictors of age at diagnosis in autism spectrum disorders: the use of multiple regression analyses and a classification tree on a clinical sample

**DOI:** 10.1007/s00787-023-02189-6

**Published:** 2023-03-18

**Authors:** Michal Hrdlicka, Tomas Urbanek, Adela Rotreklova, Aneta Kultova, Ondrej Valek, Iva Dudova

**Affiliations:** 1 Department of Child Psychiatry, Second Faculty of Medicine, Charles University, University Hospital Motol, V Uvalu 84, 15006 Prague, Czech Republic; 2 Institute of Psychology, Faculty of Arts, Masaryk University, Brno, Czech Republic; 3 Institute of Psychology, Academy of Sciences, Brno, Czech Republic; 4 Military University Hospital, Prague, Czech Republic

**Keywords:** Autism spectrum disorders, Age at diagnosis, Shared household, ADOS, Paternal age, Maternal education

## Abstract

The increasing prevalence of autism spectrum disorders (ASD) has led to worldwide interest in factors influencing the age of ASD diagnosis. Parents or caregivers of 237 ASD children (193 boys, 44 girls) diagnosed using the Autism Diagnostic Observation Schedule (ADOS) completed a simple descriptive questionnaire. The data were analyzed using the variable-centered multiple regression analysis and the person-centered classification tree method. We believed that the concurrent use of these two methods could produce robust results. The mean age at diagnosis was 5.8 ± 2.2 years (median 5.3 years). Younger ages for ASD diagnosis were predicted (using multiple regression analysis) by higher scores in the ADOS social domain, higher scores in ADOS restrictive and repetitive behaviors and interest domain, higher maternal education, and the shared household of parents. Using the classification tree method, the subgroup with the lowest mean age at diagnosis were children, in whom the summation of ADOS communication and social domain scores was ≥ 17, and paternal age at the delivery was ≥ 29 years. In contrast, the subgroup with the oldest mean age at diagnosis included children with summed ADOS communication and social domain scores < 17 and maternal education at the elementary school level. The severity of autism and maternal education played a significant role in both types of data analysis focused on age at diagnosis.

## Introduction

Autism spectrum disorders (ASD), according to The International Classification of Diseases, 10th Edition (ICD-10) [1], are neurodevelopmental conditions presenting with a range of symptoms, including impairment of communication, difficulties with social interaction, and atypical and limited patterns of behavior [2, 3].

The ASD umbrella includes autistic disorder, Asperger syndrome, pervasive developmental disorders not otherwise specified, and childhood disintegrative disorder [4]. Not only do these disorders have a significant effect on the quality of life, but they also have a considerable socioeconomic impact. The estimated global burden calculated as disability-adjusted life-years (DALY), which is an aggregation of years of life lost because of premature mortality and years lived with a disability, was 58 DALYs per 100,000 population for the autistic disorders and 53 DALYs per 100,000 for other ASDs [5].

The economic impact includes, among others, direct healthcare and special education costs and lost productivity for adults with ASD and their families/caregivers. Estimates of overall lifetime costs for individuals with ASD are in the millions of dollars, varying regionally [6].

In recent decades, we have seen a considerable increase in the reported prevalence of ASD, with recent estimates falling between 1 and 1.7%, according to studies conducted in the USA [7] and the UK [8]. No studies have specifically looked at the prevalence of autism in the Czech Republic; however, a comprehensive review of over 600 epidemiological studies worldwide (including also studies written in local languages like Arabic, Chinese, Dutch, French, etc.) did not identify regional differences in ASD prevalence, nor did it find that ethnic/cultural or socioeconomic factors had a substantial impact on prevalence [9].

It remains unclear whether the reported increase in ASD prevalence is due to an actual increase in the number of people who have autism, the result of better diagnostic tools and greater awareness, or a combination of both [10, 11]. Regardless of the cause, the increased numbers have led to a worldwide interest in studying ASD and its mechanisms.

An early autism diagnosis is essential for early intervention and treatment and enables patients to reach public support systems earlier, all of which are linked to a better prognosis, fewer autism symptoms over time, and better inclusion [10, 12]. On the other hand, delayed diagnoses are linked to increased parental stress and delays in treatments needed for better long-term trajectories [13]. Since identifying risk factors linked to delayed ASD diagnoses could potentially lead to a better diagnostic framework, it has been an area of interest in recent years.

A comprehensive review of 42 studies, written in English, published between 1990 and 2012, and indexed in PubMed, showed that higher socioeconomic status, symptom severity, and greater parental concern were associated with an earlier diagnosis [14]. The vast majority of the reviewed studies found no association between the sex of the child and age at ASD diagnosis. Furthermore, this critical and high-quality review looked at possible causes for delayed ASD diagnoses and unsurprisingly found that Asperger syndrome tends to be diagnosed at older ages than other types of ASD [14].

A 2021 Danish study supported the idea that the age at diagnosis varies relative to the subtype of ASD diagnosis and that atypical autism, Asperger syndrome, and pervasive developmental disorders not otherwise specified tend to be diagnosed at older ages [15]. This study also found an association between delayed ASD diagnosis and low parental education; however, only when compared to a group of patients that received an early autism diagnosis, not when compared to a control group with no ASD diagnosis. Lastly, this study found that being diagnosed with ADHD, language, developmental, or emotional disorders were risk factors for a delayed ASD diagnosis. The strongest association was seen in children with a previous diagnosis of ADHD [15].

In the Czech Republic, the diagnostic process of autism occurs in various settings. Two university departments (our Department of Child Psychiatry in Prague and the Department of Child Neurology in Brno) provide a diagnosis of autism during short-term hospitalizations requiring a referral from a pediatrician, child psychiatrist, neurologist, or clinical child psychologist. The process includes comprehensive assessment (e.g., psychiatric examination supplemented by specific diagnostic tools for autism, genetic examination, and an EEG). Diagnosis of autism is also provided by many but not all child psychiatrists and clinical child psychologists as outpatient care. Starting in 2016, all primary care pediatricians were obliged to begin screening all children aged 18 months for autism. All these assessments are covered by general health insurance, are free of charge, and all families are supposed to have equal access to the facilities. Besides these, services based on private payments are offered by some private providers in major towns, and we can assume they target high-income families. Regardless of the payment type, all services are generally limited by the small number of child psychiatrists with expertise in autism, which contributes to long waiting times. Due to COVID-19, waiting times now range from 1 to 2 years.

In our previous study of 160 children, we found that the ASD diagnosis age correlated negatively with maternal and paternal ages at the time of birth of the ASD child as well as with paternal and maternal education. However, it did not correlate with socioeconomic status or the number of ASD information resources available to the parents [16].

In this study, we enrolled new participants and broadened the cohort to 324 children aged 2 to 16 years. The aim of our study was to further explore the association between age at diagnosis and demographic variables, such as socioeconomic status, parental education level, age of parents, the Autism Diagnostic Observation Schedule (ADOS) scores, and intellectual disabilities.

## Methods

### Assessment

ICD-10 [1] was used to make a clinical diagnosis, and experienced clinicians with expertise in ASD diagnoses were involved in the study. A diagnosis was further supported by the age-appropriate modules from the Autism Diagnostic Observation Schedule (ADOS) [17] and/or the Autism Diagnostic Interview-Revised (ADI-R) [18]. The concept of the best estimate clinical diagnosis (BECD), by consensus of two experienced specialists, was used as the gold standard [19]. If there was disagreement between the ADI-R or ADOS diagnosis and the BECD, the latter was preferred. IQ testing was also performed. The Wechsler Preschool and Primary Scale of Intelligence, Fourth UK edition (WPPSI-IV), and the Wechsler Intelligence Scale for Children, Fourth UK edition (WISC-IV) were used for most children. For some autistic children, the Snijders-Oomen Nonverbal Intelligence Test, Revision 2, 5–7 (SON-R 2, 5–7) and the Bayley Scales of Infant and Toddler Development, Third Edition (Bayley III) were alternatively used.

Previously, we created a simple descriptive questionnaire [17] focusing on (a) the family’s general situation and (b) the family’s self-education regarding autism. For this study, we used the first part that described family information, including (1) parental ages at the initial examination and ages and at the time of birth of the ASD-affected child, (2) the educational level of the parents, (3) household situation (i.e., parents living together, or being separated/ divorced), (4) marital status, and (5) family socioeconomic status (SES). We divided the participant families into three SES subgroups based on family income; this classification was based on the Czech State legal definitions in force at the beginning of the study. See our previous publication for more details on the questionnaire [16].

Most questionnaires were completed by mothers (*N* = 205; 88.4%). Fathers completed the questionnaires in 27 cases (11.6%); missing values were noted in 5 cases.

### Sample

Responders were the parents of the 324 children referred for a diagnostic examination focused on autism at the Department of Child Psychiatry between November 2012 and June 2021. The study was approved by the Multicenter Ethics Committee of the University Hospital Motol. Parents who agreed to have their child participate in the study signed informed consent.

A diagnosis of ASD was confirmed in 237 children (73% of the referrals). The clinical characteristics of the sample are given in Table 1.

**Table 1:** Clinical characteristics of the sample

Variable	Frequency or mean/median
Gender (boys/girls)	193/44
Mean age at diagnosis (years)	5.8 ± 2.2
Median age at diagnosis (years)	5.3
Range of age at diagnosis (years)	2.2–14.8
ICD-10 diagnoses	
Childhood autism	196
Atypical autism	31
Asperger syndrome	9
Other childhood disintegrative disorders	1
Mental retardation in total (% of the sample)	148 (62.4%)
Mild	62
Moderate	59
Severe	10
Unspecified	17

*ICD-10* International Classification of Diseases, 10th Edition

Family information is displayed in Table 2.

**Table 2:** Family characteristics of the sample (n = 237)

	Mean (± SD, range) or frequency (%)
Maternal	
Age at birth of ASD child (years)	30.3 (± 5.2, 18.9–43.8)
Education	
Elementary	33 (13.9%)
High school	138 (58.2%)
University	66 (27.8%)
Paternal	
Age at birth of ASD child (years)	33.5 (± 6.2, 20.3–57.3)
Education	
Elementary	45 (19.0%)
High school	115 (48.5%)
University	68 (28.7%)
Unknown	9 (3.8%)
Family socioeconomic status	
Low	51 (21.5%)
Middle	159 (67.1%)
High	22 (9.3%)
Unknown	5 (2.1%)
Parents	
Live together	179 (75.5%)
Separated or divorced	58 (24.5%)

*SD* standard deviation, *ASD* autism spectrum disorders

### Data analysis

Statistical data analyses were performed using the statistical program R [20] and its psych library [21], in which the basic descriptive statistics of the data set and regression analyses were calculated. In addition, the rpart [22] and rpart.plot [23] libraries were used to build a machine learning-based classification tree.

A series of exploratory multiple regression analyses were initially computed to identify predictors of the age at which autism diagnoses were established. These predictors were then used in a machine-learning procedure that used sequential partitioning of the dataset.

The machine-learning algorithm consecutively divides the file into the best predictors of age at diagnosis. The division criterion is based on the ANOVA (i.e., the *F* test), meaning that the algorithm looks for the most appropriate threshold in the predictor variable, which gives the highest *F* value. This results in two branches in the tree plot (technically two subfiles of the data), one representing the earlier-diagnosed children and the other the later-diagnosed children. The algorithm then continues separately in each of these subfiles recursively, which leads to subsequent subdivisions. This process continues until no more significant predictors are left to further divide the subgroups.

At every node where the tree branches, two subgroups emerge that are significantly different from each other at the age of diagnosis. The predictor variable threshold is the highest significance of the *F* test. In each leaf of the tree, there are two numbers, i.e., the upper one is the mean age at diagnosis of its subgroup, and the lower one is the percent size of the subgroup relative to the whole sample. The division process stops when no more significant predictors are left to further divide the subgroups. The last leaves are the resulting indivisible subgroups of the original file. The darker the color, the higher the age at diagnosis the leaf represents.

## Results

In 237 children with confirmed ASD diagnosis, the ADI-R was completed in 205 cases (86.5%); of those, 199 cases (97.1%) scored positively, and only 6 cases (2.9%) were negative. The ADOS was completed for 211 children (89%); of those, results were positive in 203 children (96.2%) and negative in only eight children (3.8%). None of the children diagnosed with ASD had negative scores on both the ADI-R and ADOS.

Results of multiple regression analysis showed that younger ages at autism diagnosis were predicted by higher ADOS scores in the social domain, higher ADOS scores in the restrictive and repetitive behaviors and interest domain, higher maternal education, and both parents living in the same household; at the trend level, younger ages were associated with higher family SES (see Table 3).

**Table 3:** Predictors of age at diagnosis of autism

	Estimate	Std. error	t value	p
(Intercept)	14.056	1.105	12.716	0.000***
MAT-AGE	− 0.038	0.034	− 1.107	0.270
MAT-EDU	− *0.661*	0.258	− 2.564	0.011*
PAT-AGE	− 0.045	0.028	− 1.604	0.111
PAT-EDU	0.130	0.240	0.543	0.588
shared household	− *0.875*	0.355	− 2.469	0.014*
SES	− *0.496*	0.269	− 1.843	0.067^ǂ^
ADOS-COM	0.083	0.098	0.846	0.399
ADOS-SOC	− 0.212	0.058	− 3.667	0.000***
ADOS-RRBI	− *0.243*	0.103	− 2.36	0.019*
MR	− 0.208	0.328	− 0.633	0.528

*MAT-AGE* maternal age at birth of ASD child, *MAT-EDU* maternal education, *PAT* paternal, *SES* socioeconomic status, *ADOS-COM* communication domain of the Autism Diagnostic Observation Schedule, *ADOS-SOC* social domain of the Autism Diagnostic Observation Schedule, *ADOS-RRBI* restrictive and repetitive behaviors and interest domain of the Autism Diagnostic Observation Schedule, *MR* mental retardation

Significance codes: ***p < 0.001, **p < 0.01, *p < 0.05, ǂp < 0.1

**Fig. 1:**
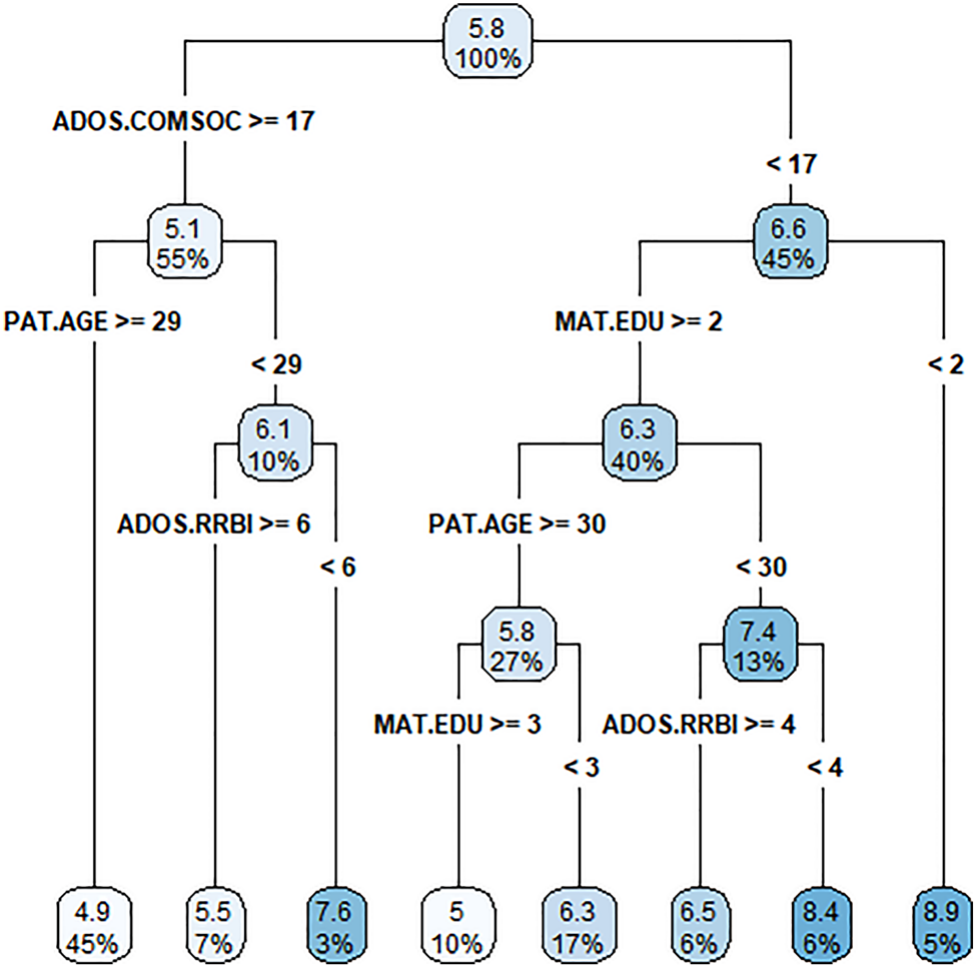
Results from the classification tree analysis of the age at diagnosis of ASD. *ADOS.COMSOC* summation of ADOS communication and social domain scores, *PAT.AGE* paternal age at birth of ASD child, *MAT.EDU* maternal education, *ADOS.RRBI* restrictive and repetitive behaviors and interest domain of the Autism Diagnostic Observation Schedule

When we applied the classification tree to the data, the youngest mean age at diagnosis (4.9 years) was found in children, in whom the summation of the ADOS communication and social domain scores was ≥ 17, and paternal age at delivery was ≥ 29 years. On the other hand, the oldest mean age at diagnosis (8.9 years) was seen in children, in whom summation of the ADOS communication and social domain scores was < 17, and maternal education was at the elementary school level (see Fig. 1).

## Discussion

Our study was only the second to use the classification tree method applied to ‘age at ASD diagnosis’ (the first study [24] was limited by the fact that it was retrospective). In contrast to regression analysis, the classification tree analysis is hierarchical, which means the classification process takes place in subsequent divisions; in our opinion, this better simulates the natural diagnostic process. While multiple regression analysis is a variable-centered approach, classification tree analysis is a person-centered approach (which is more appropriate when considering the patient’s diagnosis). Furthermore, our study was the first to simultaneously use multiple regression analysis and the classification tree method on the same data. The agreement between the two methods produced what we believe are very robust results.

The age at diagnosis in our study was calculated as a mean and median. The mean age at diagnosis of 69.6 months (5.8 years) in our study was close to, though slightly older than the findings of a 2021 systematic review and meta-analysis looking at 35 studies, which found the mean age of diagnosis was 60.48 months (5.4 years) [25]. Our result is also older than a 2020 Austrian study finding, which found the mean age at diagnosis was 46.7 months (3.9 years) [26].

The median age of 63.6 months (5.3 years) found in our study was also older than the median of 52 months (4.3 years) found in a 2018 USA study [7] as well as the median age found in a 2016 UK study of 55.2 months (4.6 years) [27] but younger than the median age of 72 months (6 years) found in a 2018 Japanese study [28]. However, it is worth noting that most studies on this topic only state the mean age at diagnosis, not the median. The disadvantage is that a relatively small number of outliers may skew the results, which is why we state both in our study.

It is also important to note that our study looked at a clinical sample. This could potentially impact the waiting times between a referral by a general practitioner and evaluation by a specialist in our department, leading to an older age at diagnosis and subsequently to the older mean and median ages seen in our study. On the other hand, having a clinical sample might have contributed to high rates of confirmed ASD diagnoses in our autism diagnostic program (73%) since the first screening was done by the referring general practitioner, child psychiatrist, or psychologist. We can only speculate that a clinical sample also results in the high severity of autism in our sample, which was reflected by a relatively high value of the first classification node, which is a summation of ADOS communication and social interaction = 17. A value of 17 and higher was present in 55% of cases (i.e., 131 cases), whereas values less than 17 were found in 45% of cases (i.e., 106 patients). For comparison, the autism cut-off is 12, and the autism spectrum cut-off is 8 in ADOS Module 2; the same cut-offs are 10 and 7 in ADOS Module 3.

Another possible explanation for our study's older age at diagnosis is that health literacy in Czechia is relatively low. According to a 2016 study, Czechia ranked penultimate compared to eight other EU countries in health literacy [29].

Multiple regression analyses and the classification tree method were used to analyze the associations between age at diagnosis, demographic variables, ADOS scores, and intellectual disability. The results of the multiple regression analysis are in line with other studies using this statistical method.

The finding that younger ages at autism diagnosis correlate with higher maternal education provides further evidence for the association between age at diagnosis and parental education [15, 30]. The link between higher scores in ADOS social domain, ADOS restrictive and repetitive behaviors and interest domain, and the younger ages at diagnosis found in our study expands on a 2014 comprehensive review, which reported that symptom severity was associated with younger ages at diagnosis [14]. The association between age at diagnosis and the ADOS social and ADOS restrictive and repetitive behaviors and interest domain found in our study also agrees with the findings of a 2021 USA study that found an association between the severity of most ASD symptoms and the age at diagnosis [24]. The finding that both parents living in the same household was associated with a younger age at diagnosis also agrees with the current literature [31].

The classification tree scheme used in our study showed that children with ADOS communication and social domain scores that summed to ≥ 17 and paternal age at delivery ≥ 29 years had the youngest mean age at diagnosis. On the other hand, the oldest mean age at diagnosis was found in children with summed ADOS communication and social domain scores < 17 and maternal education at the elementary school level.

The link between specific ADOS domains and a delayed ASD diagnosis found in our study was inconsistent with a 2018 USA study [32] and the previously mentioned 2021 Danish study [15], neither of which found such an association; however, neither of these studies used the classification tree method and therefore did not take into account maternal education and paternal age, which were key covariates in the classification tree analysis of our study.

The association between age at diagnosis and ADOS communication and social domain scores was consistent with the previously mentioned USA study [24], which used factor analysis and was the first study prior to ours that applied the classification tree methodology. This study found an association between younger ages at diagnosis and deficits in communication skills, i.e., delayed language, absence of gestures, and responding to one’s name. Additionally, the study found the association even more robust than the one between age at diagnosis and overall symptom severity. However, like most other studies, this study did not consider maternal education and paternal age in their classification tree analysis.

In contrast to several US studies [33–36] and a meta-analysis of 42 studies by Daniels and Mandell [14], family socioeconomic status was not found to be a significant predictor in our study [it was significant only at the trend level (*p* = 0.067)]. Our SES results agree with the largest meta-analysis on the topic by Elsabbagh et al. [9], which involved over 600 studies (including studies in local languages).

### Limitations

Possible limitations of our study include the fact that our study looked at a clinical sample, which could have caused the age at diagnosis in our study to be older than in population samples. The second limitation is that we did not consider comorbidities other than mental retardation, which could also influence the age at diagnosis. The third limitation is that we focused our analysis on global measures of autism as well as on family and socioeconomic circumstances rather than on particular autistic symptoms. Thus, we did not examine language development using a specific diagnostic tool other than the communication domain of ADOS, which did not influence the age at diagnosis in the multiple regression analysis (see Table 3); however, it was significant, in summation with the ADOS social domain, in classification tree analysis (see Fig. 1). The fourth limitation was related to the fact that for this research, we did not collect and analyze information regarding who referred the children to our autism diagnostic program. Therefore, we cannot confirm the Austrian findings that a referral by a pediatrician was associated with an earlier diagnosis [26]. Our results could have also been influenced by our sample, including only children, thereby excluding those diagnosed with autism in adulthood. However, autism diagnoses in adulthood in the Czech Republic are rare due to limited services for adult autistic subjects, although new data from the UK indicates that diagnosis rates among adults, females, and higher-functioning individuals are rising [37].

## Conclusion

The severity of autism symptoms and maternal education impacted the age at diagnosis in both types of data analysis used in our study (i.e., multiple regression analysis and classification tree analysis).

## Data Availability

The data presented in this study is available on request from the corresponding author. The data is not publicly available due to privacy reasons.
